# Comparison of Biochemical and Physiological Properties of Two Brassica Microgreens Cultivated in Two Growth Systems

**DOI:** 10.3390/plants15030465

**Published:** 2026-02-02

**Authors:** Michele Ciriello, Christophe El-Nakhel, Giovanna Marta Fusco, Petronia Carillo, Youssef Rouphael, Giandomenico Corrado

**Affiliations:** 1 Department of Agricultural Sciences, University of Naples Federico II, 80055 Portici, Italy; michele.ciriello@unina.it (M.C.); christophe.elnakhel@unina.it (C.E.-N.); 2 Department of Environmental, Biological and Pharmaceutical Sciences and Technologies, University of Campania “Luigi Vanvitelli”, 81100 Caserta, Italy; giovannamarta.fusco@unicampania.it (G.M.F.); petronia.carillo@unicampania.it (P.C.)

**Keywords:** substrate, unheated greenhouse, soilless cultivation, secondary metabolites, functional food

## Abstract

Microgreens are promising crops for low-input systems, but the roles of species traits and root environments under uncontrolled greenhouse conditions are not yet fully addressed. In this study, mibuna and pak choi were evaluated in the presence or absence of substrate to clarify how genetic and physical factors shape growth and biochemical composition. Clear species-dependent differences emerged. Pak choi showed higher constitutive levels of chlorophyll a, anthocyanins, and key osmotic ions, and these features contributed to a more hydrated tissue profile. Mibuna instead exhibited a higher dry matter content, approximately 7% compared with 5.86% in pak choi, and a lighter canopy. The use of peat markedly improved overall growth. Fresh yield increased by more than 50%, and dry yield increased by almost 48% compared with the substrate-free system. Peat also enhanced organic acid metabolism, with malate and citrate increasing by 277 and 102%, respectively. Despite such differences, nitrate concentrations remained low and within safe limits for leafy vegetables in all treatments. The results indicate that species identity and the cultivation system significantly shaped overall microgreen performance under low-input conditions. Understanding these drivers provides a foundation for optimizing production while maintaining desirable nutritional traits.

## 1. Introduction

Consumers increasingly seek fresh, unprocessed, and wholesome foods rich in bioactive compounds to support healthier lifestyles [1,2]. In light of these demands, microgreens, young plants harvested before the development of the first two true leaves, emerge as a valuable dietary component for improving and diversifying eating habits [3]. Initially valued mainly as colorful garnishes, microgreens have evolved into a widely consumed functional food [4]. They are ideal for those seeking a healthy diet, as well as for vegetarians, vegans, and raw foodists, owing to their ability to diversify and enrich the diet with a vast array of varieties [5,6]. Their low-calorie content, combined with high vitamin and mineral levels, makes them suitable for low-calorie diets [7]. Notably, the early harvesting stage that characterizes microgreens usually results in a higher concentration of the most sought-after bioactive compounds (polyphenols, vitamins, microelements, etc.) [8].

Microgreens encompass diverse botanical families, including Brassicaceae, Asteraceae, Apiaceae, Amaryllidaceae, and Amaranthaceae [4,6]. This botanical diversity is accompanied by a growing popularity and interest, substantiated by economic trends. In 2025, the turnover related to the production and consumption of microgreens reached USD 17 billion; in the United States alone, the compound annual growth rate (CAGR) has grown by approximately 10% over the last five years (2020–2025) [4]. In addition to their nutritional qualities, other often overlooked operational advantages should be considered when placing microgreens among the most interesting foods of the future. The short cultivation cycle (7–28 days from sowing) allows for multiple production cycles per year, [9]. In urban agriculture, microgreen production represents an innovative, efficient, and sustainable agricultural option, as the need for resources such as water, nutrients, and land is significantly lower than that required for traditional crops [10]. Furthermore, the short production cycle, characterized by a very narrow window between sowing and harvest, greatly reduces or typically eliminates the need for synthetic pesticides, usually resulting in a safer, residue-free food source [11].

Although the literature consistently recommends controlled environments, regions with favorable climates can successfully adopt microgreen production in uncontrolled greenhouses, thereby reducing costs and energy dependence [12]. Although in recent years, numerous scientific studies have investigated the effects of the different pre-harvest factors involved in the production of microgreens [12,13], standardized protocols for optimizing yield across diverse species and growing systems remain largely unestablished. Excluding the influence of genetic diversity, the growth substrate represents one of the most critical production factors impacting microgreen biomass accumulation [14,15,16]. The study conducted by Kyriacou et al. [17] on the cultivation of coriander, kohlrabi, and pak choi microgreens on substrates of different types and origins (agave fiber, capillary mat, cellulose sponge, coconut fiber, and peat moss) highlights that the ideal substrate for ensuring satisfactory yields must combine physical characteristics that maintain a favorable balance between water retention and aeration during and between irrigation. The same authors observed that, compared to peat, which is the ideal substrate in terms of physical characteristics, the use of synthetic substrates (such as sponge and capillary mats) was limited by low air capacity (14.5% *v*/*v* on average), which would require reduced and very frequent irrigation volumes to ensure adequate aeration of the roots. However, considering the short growing cycle, non-biodegradability, and high costs, the choice of these substrates could significantly impact the sustainability of the entire production chain [18].

The spread of closed-loop hydroponics has been a turning point for leafy vegetable production. Several authors have pointed out that the transition from soil to soilless cultivation not only guarantees better yields per unit but also significantly increases resource efficiency while reducing labor costs [10,19,20]. Even though hydroponics is the most widely used cultivation method for microgreens, substrate remains an imperative element. El-Nakhel et al. [21], using specific containers that reproduce cultivation in a floating system, evaluated the possibility of growing microgreens without substrate. Despite the results showing good adaptability of two species (*Daucus carota* L. and *Anethum graveolens* L.) to the innovative cultivation system, it would be appropriate to assess what can be achieved under the same conditions but using a substrate. Although substrate use is currently regarded as essential for commercial microgreen production, we hypothesized that a simplified, static floating system could still support satisfactory growth by leveraging the short cultivation cycle and the plants’ reliance on endogenous seed reserves. Furthermore, we postulated that the absence of a physical buffer might induce mild stress, potentially altering secondary metabolite profiles in a compound- and species-specific manner, without compromising yield. Based on these premises, the rationale of this study was to contrast the ‘agronomic standard’ (peat-based cultivation) against a ‘sustainable alternative’ (substrate-free floating system). The primary objective was to determine if the substrate-free approach could maintain acceptable yield and quality levels, thereby offering a viable low-input strategy for greenhouse production.

To test this, we compared the productive and biochemical responses of two distinct *Brassica* genotypes, mibuna and pak choi, grown with and without substrate under uncontrolled conditions. These species were selected not only for their economic importance within the Brassicaceae family but also to evaluate how their contrasting canopy architectures and growth rates adapt to the specific root-zone conditions (i.e., high water availability) of substrate-free systems. Understanding these interactions is useful for assessing the feasibility of innovative, low-input methods that omit the use of substrate.

## 2. Results

### 2.1. Biometric Parameters and Yield

**Figure 1:**
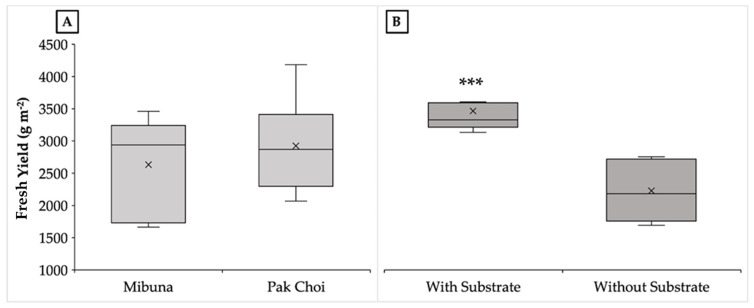
Effect of species (**A**) and growth system (**B**) on fresh yield. Comparisons between means were performed using Student’s *t*-test. Asterisks indicate significant differences according to Student’s *t*-test (*** *p* < 0.001).

Contrary to the findings of Kyriacou et al. [22], who reported significant yield differences between mibuna (2.63 kg m^−2^) and pak choi (2.45 kg m^−2^) grown on peat, our experiment showed no significant differences in fresh yield, dry yield, or hypocotyl height between the two species, regardless of the cultivation system (Figure 1 and Table 1).

On the contrary, the same parameters were significantly influenced by the presence or absence of substrate. Specifically, microgreens grown on peat guaranteed a fresh and dry yield 54.3 and 47.5% higher than that recorded in the absence of substrate. Similarly, regardless of species, microgreens grown on peat-based substrate were characterized by greater height (7.49 cm), while no difference was observed in terms of dry matter percentage (on average—6.4%). As with the colorimetric parameters, the dry matter content was significantly influenced exclusively by the species (Table 1). Specifically, compared to pak choi microgreens, mibuna had a higher percentage of dry matter (7.0%).

### 2.2. Colorimetric Attributes and Pigments

Compared to pak choi microgreens, mibuna was characterized by greater brightness and greenness, as indicated by its higher L and a* values, respectively (Table 2). On the other hand, pak choi microgreens, regardless of the cultivation system, displayed lower b* (11.05) and Chroma (11.16) values.

As shown in Table 3, all pigment-related traits in microgreens were influenced only by the main effect of species. Although total chlorophyll content did not differ significantly between species (on average—2.37 µg g^−1^ DW), pak choi microgreens contained higher chlorophyll a (1.74 µg g^−1^ DW). In line with the main colorimetric indices measured and presented in Table 2, pak choi microgreens had an anthocyanin content more than one hundred times higher than that of mibuna microgreens (Table 3). In contrast, carotenoids and total polyphenols were not significantly influenced by any of the experimental factors considered (Table 3).

### 2.3. Mineral and Organic Acid Content

Table 4 and Table 5 show the mineral profiles and organic acid content (malate, oxalate, and citrate) of the two species of microgreens grown with or without substrate. Even though the use of substrate did not cause any significant differences in nitrate content, the microgreens grown in a floating system (without substrate) had a numerically lower nitrate content (456.7 mg kg^−1^ f.w) than those grown on substrate (519.6 mg kg^−1^ f.w) (Table 4).

Conversely, microgreens grown on peat-based substrate had a higher content of SO_4_^2−^ (21.67 g kg^−1^ DW), K^+^(16.6 g kg^−1^ DW), and Ca^2+^ (13.94 g kg^−1^ DW). No significant effects were observed for Cl^−^, PO_4_^3−^, and Mg^2+^ (Table 4). Similarly, compared to the floating system (without substrate), the use of peat increased malate and citrate contents by 277.1% and 102.2%, respectively (Table 5). No difference was found in the oxalate content. Regardless of the growing system, pak choi microgreens accumulated higher content of Cl^−^ (3.02 g kg^−1^ DW), Na^+^ (1.34 g kg^−1^ DW), PO_4_^3−^ (21.86 g kg^−1^ DW), K^+^ (16.28 g kg^−1^ DW), and Mg^2+^ (3.33 g kg^−1^ DW) than mibuna. No significant species-related difference was observed for NO_3_^−^, SO_4_^2−^, and Ca^2+^ content (Table 5).

### 2.4. Principal Component Analysis

**Figure 2:**
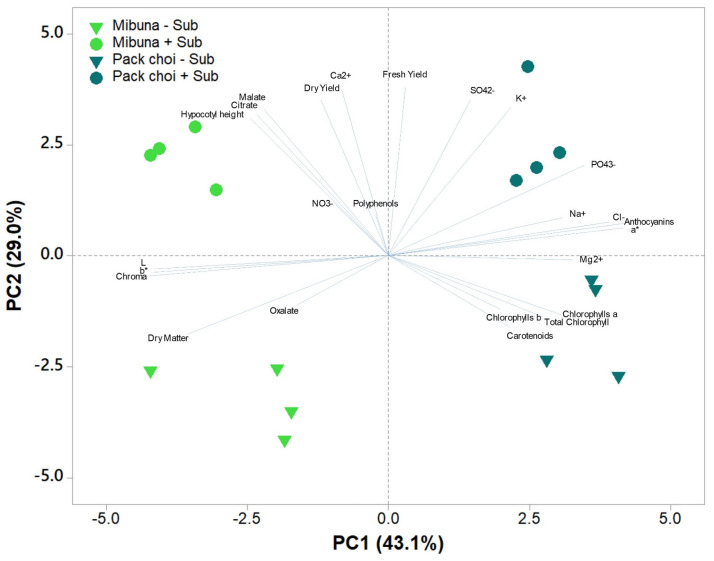
Principal component analysis (PCA) of biometric traits, color parameters, mineral composition, photosynthetic pigments, organic acids, and nutraceutical compounds of mibuna and pak choi microgreens grown with (+Sub) or without (–Sub) substrate.

To obtain an integrated interpretation of growth performance, color attributes, mineral composition, and nutritional traits of mibuna and pak choi microgreens grown with or without substrate, a principal component analysis (PCA) was performed, including all measured variables. The first four principal components (PCs) showed eigenvalues greater than 1 and together explained 87.6% of the total variance, with PC1, PC2, PC3, and PC4 accounting for 43.1%, 29.0%, 10.1% and 5.5%, respectively (Figure 2). PC1 clearly separated the two microgreen species. Pak choi samples were positioned on the positive side of this axis and were associated with higher concentrations of minerals (K^+^, PO_4_^3−^, Na^+^, Cl^−^, and Mg^2+^), elevated chlorophylls and carotenoids, and greater fresh and dry yields. This result reflects the generally richer biochemical and mineral profile of pak choi. In contrast, mibuna microgreens were located on the negative side of PC1, in association with traits such as higher L*, b*, and Chroma values, greater dry matter content, and increased oxalate levels, which correspond to their lighter coloration and structurally denser tissues. PC2 mainly described differences attributable to the cultivation system. Microgreens grown with substrate were distributed along the positive side of this component, driven by the strong influence of malate, citrate, SO_4_^2−^, and Ca^2+^, together with higher fresh and dry yields. By contrast, samples grown without substrate clustered on the negative side of PC2, in correspondence with higher chlorophyll and carotenoid concentrations and increased dry matter, indicating that pigment accumulation was favored in the floating system.

## 3. Discussion

An optimal management of the different pre-harvest factors plays a crucial role in achieving specific objectives in terms of both yield and sustainability of the production process [15]. Considering that microgreens are generally sold fresh, achieving a satisfactory yield is a major challenge for producers [12]. The results of our study show that both growth parameters and qualitative attributes were mainly influenced by the main effects of species and substrate, with limited evidence of mutual interaction between the two factors. This clearly indicates that plant species and substrate use were the dominant drivers of variation, and, in general, the species used in this experiment responded similarly to changes in growth conditions associated with the presence or absence of substrate.

This interpretation is corroborated by the PCA, which synthesizes the results across the different analytical approaches. The multivariate analysis confirmed that species identity and substrate presence were the primary drivers of the phenotypic and biochemical profiles, while their interaction contributed marginally to the observed variability.

The absence of a significant yield difference between mibuna and pak choi stands in contrast to the established literature, where genotypic influence is typically the primary determinant of variation [23,24,25]. While the observed uniformity may stem from the close phylogenetic relationship between these specific *Brassica* cultivars, it is equally plausible that the fluctuating environmental conditions of the unheated greenhouse masked the potential for species-specific physiological expression. This hypothesis is supported by the findings of Kyriacou et al. [22], who observed distinct yield hierarchies among four *Brassica* species (including the two tested here) when cultivated under the strictly regulated environmental parameters of a growth chamber. Although few studies have explored the possibility of growing microgreens in uncontrolled environments, the production results obtained in our study are very similar to those reported for the same species under controlled conditions [26]. Although achieving increasingly high production standards is an absolute priority in the world of microgreens, the possibility of growing them without relying on external inputs, such as uncontrolled greenhouses, could be a considerable advantage, especially in areas with favorable climatic conditions. This argument also applies to substrate-free production, given that substrates are often costly and non-renewable [15,21]. While our results highlight the real possibility of efficiently producing microgreens in uncontrolled conditions, the use of a production system characterized by the complete absence of substrate did not allow us to achieve yields comparable to those obtained on peat. A comparison of organic substrates shows that the better production performance recorded in microgreens grown on peat may be partly linked to the better chemical and nutritional composition of the substrate itself [27,28,29]. However, considering that throughout the experiment, both the microgreens grown on peat and those grown without substrate were fertirrigated with a nutrient solution containing all the essential macro and microelements, we can hypothesize that the positive effects of peat are not strictly and/or uniquely linked to the chemical composition of the substrate. Conversely, microgreens grown on peat may have benefited from a more optimal rooting environment attributable to the physical properties of the substrate. The peat-based substrates likely provided better root aeration and improved mechanical support. In contrast, the constant submersion of roots in the substrate-free floating system may result in root zone hypoxia, which is a known potential limiting factor in static hydroponic systems [30,31]. Reduced oxygen availability limits root respiration and nutrient uptake, thereby contributing to the lower fresh and dry yields observed compared to the peat treatment. Th presence of the substrate may have created a thermal buffer that reduced strong temperature fluctuations at the root level. [30,31]. These temperature fluctuations, amplified in the absence of substrate, may have been particularly detrimental in our uncontrolled environment.

The lower yield of microgreens grown without substrate is partly confirmed by a lower dry yield and hypocotyl height. As pointed out by Dantas et al. [32], the height of microgreens is an important product characteristic, and the presence of peat, regardless of the species considered, favored the production of bigger seedlings. Even though it is widely recognized in the literature that different growing strategies, like the choice of substrate, markedly influence the dry matter content in microgreens [33,34], in our study, variations in this parameter were attributable exclusively to the genetic diversity of the two species tested. Ordinarily, the cultivation of mature plants in hydroponic systems, such as the one used in our experiment, encourages plants to accumulate greater amounts of water due to a constant supply of nutrient solution [35]. Probably during this phenophase of the plant, the regulation of transpiration activity could be less influenced by the cultivation system, contrary to what has been described for mature counterparts. On the contrary, again with regard to biometric parameters, it is precisely the percentage of dry matter that is the main discriminating factor between the two species. Specifically, mibuna had a lower water content, which is not insignificant, as it is precisely the high water content that limits the storage and handling of microgreens [36]. This difference is consistent with the ionic composition of the two species, as pak choi displayed higher concentrations of K^+^, Na^+,^ and Cl^−^, which are key osmotic regulators capable of lowering cellular water potential and promoting greater tissue hydration [26].

Another critical aspect for the marketability of microgreens is visual perception. The colorimetric characteristics of microgreens influence consumer perception, directly affecting the appeal of the product and, consequently, its market value [37]. As with the percentage of dry matter, the colorimetric parameters were also found to be influenced exclusively by the species. The lack of variation in the colorimetric parameters in the two cultivation scenarios tested (with substrate vs. without substrate) is justified by the unchanged content of pigments such as chlorophyll and carotenoids. In addition to their biochemical role in plant physiology, these pigments are also important for the human body, thanks to their antioxidant power and ability to trigger synergistic relationships with other bioactive compounds [38]. The color difference between the two species was obviously attributable to the higher recorded biosynthesis of anthocyanins in pak choi. In addition to having a significant impact on color components, the higher anthocyanin content provides pak choi with superior nutraceutical characteristics, considering the positive effect of these molecules on human health [39].

Several studies on microgreens have hypothesized that the high antioxidant potential of microgreens is linked to the rich presence of phenolic compounds [40,41]. The biosynthesis of these valuable secondary metabolites appears to be strongly influenced by the choice of genotype and the specific growing conditions used. However, as with yield, phenolic content was not influenced by the use of substrate, confirming the hypothesis that uncontrolled growing conditions could mask the effects of other factors, such as substrate choice. In addition to their interesting biochemical composition, microgreens are considered a good source of minerals [15,42]. In sharp contrast to the most commonly available leafy vegetables, microgreens can be safely consumed, as their nitrate concentrations is generally very low. Our results showed that, regardless of growing conditions, the concentration of this anti-nutrient ranged from 470.8 to 505.5 mg kg^−1^ of fresh weight, confirming their status as non-nitrate accumulators [43], well below current EU limits for leafy vegetables intended for fresh consumption (i.e., 2000–3000 mg kg^−1^ FW for lettuce and up to 3500 mg kg^−1^ FW for spinach according to Reg. EU 1258/2011). In partial agreement with the findings of Bulgari et al. [15], nitrate levels were not influenced by species or substrate use. Cultivation on peat, on the other hand, significantly influenced the content of K^+^, SO_4_^2-^, and Ca^2+^, nutrients essential for plant growth and development [44,45]. The higher concentration of key nutrients, in addition to providing microgreens grown on peat a better nutritional value, would partly justify the better production performance described above. The higher calcium content in microgreens grown on peat could be directly related to the height of the microgreens, which would consequently have affected the need for mechanical support as a result of a more developed “stem” component. Finally, the marked increases in malate and citrate observed in peat-grown microgreens indicate a more active organic acid metabolism. This response is consistent with the enhanced root aeration and improved respiratory efficiency typically associated with well-structured substrates, which promote greater flux through the tricarboxylic acid (TCA) cycle [46]. Higher malate and citrate concentrations may also shape sensory traits, with malate contributing to acidity and freshness and citrate imparting a sharper sour note, both of which are relevant descriptors in Brassica microgreens [47]. Beyond their sensory impact, organic acids play key roles in intracellular charge balance and cation homeostasis, and their accumulation can facilitate Ca^2+^ and other nutrient stabilization through chelation and pH-modulated mechanisms [48]. Overall, these trends suggest that peat not only enhanced biomass production but also created a metabolic context favorable to organic acid synthesis and coordination.

## 4. Materials and Methods

### 4.1. Growth Conditions, Plant Material, and Experimental Design

**Figure 3:**
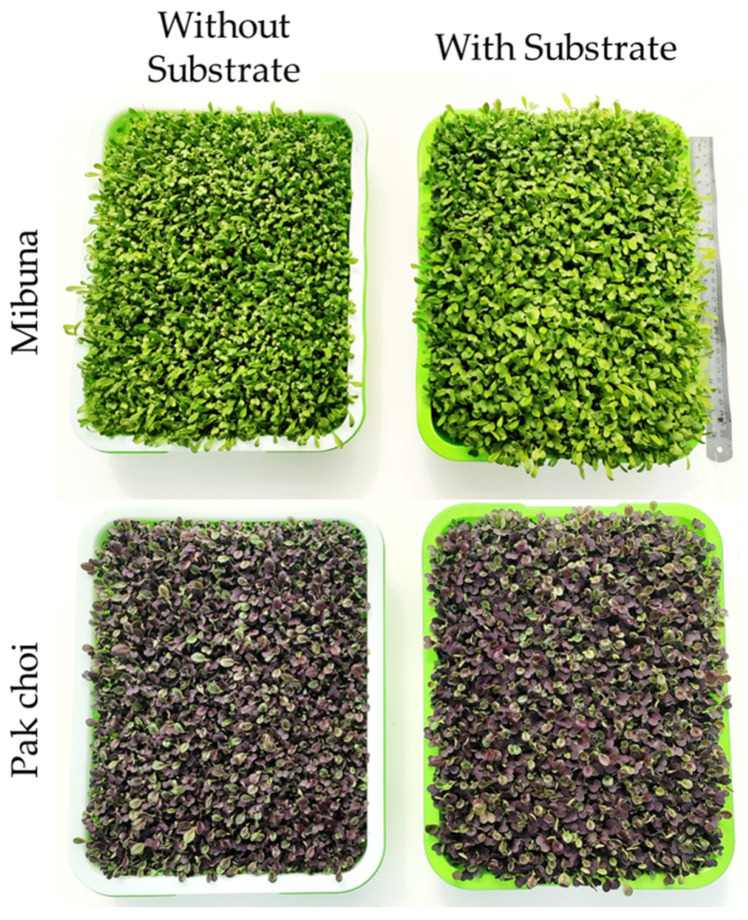
Visual comparison of mibuna (**top row**) and pak choi (**bottom row**) microgreens cultivated in a substrate-free floating system (**left column**) versus a peat-based substrate system (**right column**). Images were acquired at the commercial harvest stage (16 days after sowing).

The experimental trial comparing the yield response of two species of microgreens grown with and without the use of substrate was conducted in an unheated greenhouse at the Department of Agriculture of the Federico II University of Naples (Portici, Naples; latitude 40°49′11′64 N, longitude 14°20′28′68 E, 29 m above sea level). During the experiment, the average air temperature was 23 °C (range: 12–31 °C), and relative humidity ranged from 55% (day) to 80% (night). The average photosynthetic photon flux density (PPFD) between 7:00 a.m. and 5:30 p.m. was approximately 600 µmol m^−2^ s^−1^. On 1 April 2024, seeds of mibuna (*Brassica rapa* L. subsp. nipposinica; Micro Splits, CN Seeds Ltd., Pymoor, Ely, Cambridgeshire, United Kingdom) and pak choi (*Brassica rapa* L. subsp. chinensis; Micro Splits, CN Seeds Ltd., Pymoor, Ely, Cambridgeshire, United Kingdom) were sown in plastic trays with a surface area of 588 cm^2^. For both species, a sowing density of 5 seeds cm^−2^ was used. The microgreens trays were arranged according to a randomized two-factor experimental design, in which the factors were the different growth systems (with substrate vs. without substrate) and two species of microgreens (mibuna and pak choi). Each treatment was replicated four times (n = 4; 16 experimental units total), and each replication consisted of a single tray with a surface area of 588 cm^2^ (Figure 3).

**Figure 4:**
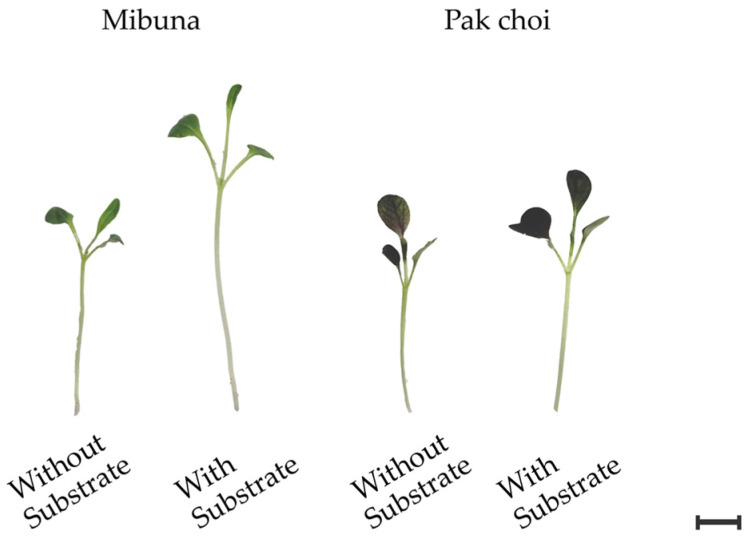
Representative morphological comparison of mibuna and pak choi microgreens at harvest (16 days after sowing). The image displays the impact of the growth system (substrate-free vs. peat-based substrate) on seedling development and hypocotyl elongation. Scale bar = 1 cm.

For treatments where no substrate was used, the plastic tray had holes across its entire surface (588 cm^2^) and was placed inside a plastic tank containing a maximum volume of 1 L. For treatments with substrate [peat mixture with pH 5.48 and electrical conductivity (EC) 282μS cm^−1^; Floragard Vertriebs-GmbH, Oldenburg, Germany], the same unperforated trays were filled with 1 L of peat. The composition and concentration of the macroelements constituting the substrate used are described in detail by Pannico et al. [49] A quarter-strength modified Hoagland nutrient solution was prepared from deionized water (EC 0.03 dS m^−1^; pH 6.2), containing 2.0 mM NO_3_^−^, 0.25 mM S, 0.20 mM P, 0.62 mM K, 0.75 mM Ca, 0.17 mM Mg, 0.25 mM NH_4_^+^, 20 μM Fe, 9 μM Mn, 0.3 μM Cu, 1.6 μM Zn, 20 μM B, and 0.3 μM Mo, with a final EC of 0.4 ± 0.05 dS m^−1^. This solution was used for all treatments. In peat-based treatments, the nutrient solution was distributed daily using laboratory beakers, whereas in substrate-free treatments, fresh nutrient solution was added every two days, restoring the original volume of 1 L. All treatments were harvested 16 days after sowing (Figure 4).

### 4.2. Harvest, Biometric, and Colorimetric Parameters

Sixteen DAS, mibuna, and pak choi microgreens were harvested using sterilized scissors at the tray level. Fresh yield was expressed in g m^−2^. Twenty microgreens representative of each replicate were selected for hypocotyl height determination. A portion of the harvested fresh microgreens was placed in a ventilated oven at 65 °C for 72 h to determine dry weight (expressed as g m^−2^), and the percentage of dry matter (DM) was then calculated. The dried material was ground (MF10.1 Wiley laboratory mill, IKA^®^, Staufen im Breisgau, Baden-Württemberg, Germany), sieved, and used to determine its mineral content. The remaining fresh material was shock-frozen in liquid nitrogen, freeze-dried, and stored at −80 °C for further analysis. Before harvesting, the color of the microgreens’ canopy was determined at ten different points on each tray using a Minolta CR-300 colorimeter (Minolta Camera Co. Ltd., Osaka, Japan). The instrument used was appropriately calibrated before colorimetric measurements were taken. Specifically, the measurements were obtained using the parameters of the CIELAB (Commission Internationale de l’Éclairage) color space.

### 4.3. Determination of Ion and Organic Acid Content

As described in detail by Formisano et al. [50], the concentration of anions (NO_3_^−^, SO_4_^2−^, PO_4_^3−^, Cl^−^), cations (K^+^, Ca^2+^, Mg^2+^, Na^+^), and organic acids (malate, citrate, and oxalate) was determined by ion chromatography coupled with an electrical conductivity detector (ICS-3000, Dionex, Sunnyvale, CA, USA). After comparing the peak areas of the samples with those of the individual standards, ion integration and quantification were performed using Chromeleon™ 6.8 Chromatography data system software (Dionex, Sunnyvale, CA, USA). Except for nitrates expressed as mg kg^−1^ on fresh weight (FW), all other ions were expressed as g kg^−1^ on dry weight (DW).

### 4.4. Photosynthetic Pigments and Phenolic Compounds

The analysis of photosynthetic pigment content (chlorophyll a and b, and carotenoids) was determined according to Wellburn [51]. Lyophilized samples (10 mg) were extracted in 1 mL of methanol and centrifuged at 13,500 rpm for 5 min. Absorbance of the extracts at 665, 652, and 470 nm was recorded using a Synergy HT spectrophotometer (BioTek Instruments, Bad Friedrichshall, Germany) and used to calculate chlorophyll a, chlorophyll b, and total carotenoids, expressed as µg g^−1^ DW. Total polyphenols were quantified according to Singleton et al. [52], with minor modifications. Lyophilized leaf tissue (30 mg) was extracted in 700 µL of 60% (*v*/*v*) methanol. An aliquot (45 µL) was mixed with 115 µL of Folin–Ciocalteu reagent (1:4, *v*/*v*), and after 6 min, 650 µL of 3% (*v*/*v*) sodium carbonate was added. Samples were incubated at room temperature for 90 min. Absorbance at 760 nm was measured with a Synergy HT microplate reader (BioTek Instruments), and polyphenols were quantified against a gallic acid calibration curve and expressed as mg GAE g^−1^ DW. Total anthocyanins were determined as described by Nicastro et al. [53], with some modifications. Lyophilized samples (40 mg) were extracted twice with 180 µL of 40% (*v*/*v*) ethanol on ice for 20 min and centrifuged (14,000 rpm, 10 min). Combined supernatants (150 µL) were mixed with either 75 µL of 25 mM KCl buffer (pH 1.0) or 75 µL of 400 mM sodium acetate buffer (pH 4.5). Absorbance at 520 and 700 nm was recorded with a Synergy HT spectrophotometer (BioTek Instruments). Anthocyanin content was expressed as mg cyanidin-3-glucoside equivalents (C3G eq) g^−1^ DW.

### 4.5. Statistical Analysis

All analyses were performed in quadruplicate. All experimental data were subjected to two-way analysis of variance using IBM SPSS Statistics version 30.0 (SPSS Inc., Chicago, IL, USA). The main effects [microgreen species (MS) and growing system (GS)] were compared using Student’s t-test. For the interaction of the factors considered (MS × GS), statistically significant differences were determined using the Tukey–Kramer HSD test at the level of *p* < 0.05. The Principal Component Analysis (PCA) was carried out using Minitab 18 (Minitab Inc., State College, PA, USA).

## 5. Conclusions

This work demonstrates that species identity and the physical structure of the root environment are main factors shaping microgreen performance under low-input, uncontrolled greenhouse conditions. Mibuna and pak choi expressed clearly differentiated physiological behaviors, reflecting intrinsic differences in pigment composition, osmotic adjustment, and tissue organization, which in turn influenced their functional and nutritional profiles. At the same time, peat appeared to have provided a more favorable rhizosphere than the substrate-free system, potentially ensuring more stable root conditions and supporting the metabolic processes associated with organic acid turnover and mineral homeostasis. These combined effects highlight that efficient microgreen production outside controlled environments depends on the careful interplay between species-specific traits and the physical properties of the growth medium. A refined integration of both aspects emerges as a key requirement for maintaining product quality and physiological integrity in simplified cultivation systems.

To improve the performance of substrate-free microgreens, future research should focus on bridging the yield gap compared to conventional systems. Potential strategies include increasing the nutrient solution volume in static hydroponic systems to improve oxygenation and buffer against thermal fluctuations. Additionally, the implementation of active oxygenation or dynamic nutrient replenishment synchronized with root growth should be evaluated.

## Figures and Tables

**Table 1:** Effect of species and growing system on biometric parameters.

Treatments	Dry Yield	Dry Matter	Hypocotyl Height
	g m^−2^	%	cm
Microgreen species (MS)			
Mibuna	181.91 ± 16.3	7.0 ± 0.15 a	6.92 ± 0.42
Pak Choi	170.91 ± 16	5.86 ± 0.2 b	5.88 ± 0.38
Growing system (GS)			
With Substrate	210.28 ± 9.71 a	6.23 ± 0.19	7.49 ± 0.21 a
Without Substrate	142.54 ± 7.7 b	6.62 ± 0.28	5.31 ± 0.2 b
MS	ns	***	ns
GS	***	ns	***
MS × GS	ns	ns	ns

ns and *** non-significant or significant at *p* ≤ 0.001, respectively. Different letters within each column indicate significant mean differences according to Student’s *t*-test (*p* = 0.05). All data are expressed as mean ± standard error, n = 4.

**Table 2:** Effect of species and growing system on colorimetric parameters.

Treatments	L	a*	b*	Chroma
Microgreen species (MS)				
Mibuna	40.36 ± 0.53 a	−15.73 ± 0.38 a	29.81 ± 0.6 a	33.73 ± 0.56 a
Pak Choi	27.84 ± 1.36 b	−0.4 ± 1.89 b	11.05 ± 2.66 b	11.16 ± 3.16 b
Growing system (GS)				
With Substrate	34.91 ± 2.32	−8.3 ± 2.85	21.16 ± 3.82	23.09 ± 4.52
Without Substrate	33.3 ± 2.52	−7.83 ± 3.02	19.71 ± 3.34	21.8 ± 4.08
MS	***	***	***	***
GS	ns	ns	ns	ns
MS × GS	ns	ns	ns	ns

Legend: ns and *** non-significant or significant at *p* ≤ 0.001, respectively. Different letters within each column indicate significant mean differences according to the Student’s *t*-test (*p* = 0.05). All data are expressed as mean ± standard error, n = 4.

**Table 3:** Effect of species and growing system on pigment and phenol content.

Treatments	Chlorophylls a	Chlorophylls b	Total Chlorophyll	Carotenoids	Anthocyanins	Polyphenols
	µg g^−1^ DW	µg g^−1^ DW	µg g^−1^ DW	µg g^−1^ DW	mg C3G eq g^−1^ DW	mg GAE g^−1^ DW
Microgreen species (MS)						
Mibuna	1.43 ± 0.11 b	0.68 ± 0.12	2.12 ± 0.19	0.41 ± 0.04	0.01 ± 0.002 b	28.97 ± 0.64
Pak Choi	1.74 ± 0.11 a	0.88 ± 0.11	2.62 ± 0.2	0.47 ± 0.04	1.34 ± 0.161 a	28.94 ± 0.63
Growing system (GS)						
With Substrate	1.5 ± 0.1	0.74 ± 0.08	2.24 ± 0.18	0.41 ± 0.03	0.66 ± 0.24	29.36 ± 0.29
Without Substrate	1.68 ± 0.1	0.82 ± 0.13	2.5 ± 0.21	0.46 ± 0.04	0.69 ± 0.26	28.55 ± 0.85
MS	*	ns	ns	ns	***	ns
GS	ns	ns	ns	ns	ns	ns
MS × GS	ns	ns	ns	ns	ns	ns

ns, *, and *** non-significant or significant at *p* ≤ 0.05 and 0.001, respectively. Different letters within each column indicate significant mean differences according to Student’s *t*-test (*p* = 0.05). All data are expressed as mean ± standard error, n = 4.

**Table 4:** Effect of species and growing system on ionic content.

Treatments	NO_3_^−^	Cl^−^	SO_4_^2−^	PO_4_^3−^	Na^+^	K^+^	Mg^2+^	Ca^2+^
	mg kg^−1^ FW	g kg^−1^ DW						
Microgreen species (MS)								
Mibuna	505.54 ± 55.7	2.43 ± 0.04 b	15.16 ± 1.71	14.33 ± 0.98 b	1.1 ± 0.04 b	11.63 ± 0.91 b	2.7 ± 0.08 b	9.56 ± 1.85
Pak Choi	470.77 ± 49.07	3.02 ± 0.07 a	19.91 ± 2.01	21.86 ± 1.42 a	1.34 ± 0.04 a	16.28 ± 1.3 a	3.33 ± 0.09 a	9.33 ± 1.81
Growing system (GS)								
With Substrate	519.61 ± 52.04	2.72 ± 0.14	21.67 ± 1.13 a	19.49 ± 1.22	1.22 ± 0.07	16.6 ± 0.99 a	2.88 ± 0.07	13.94 ± 0.33 a
Without Substrate	456.71 ± 36.33	2.74 ± 0.1	13.4 ± 1.01 b	16.71 ± 1.85	1.22 ± 0.05	11.31 ± 0.84 b	3.15 ± 0.21	4.95 ± 0.17 b
MS	ns	***	ns	***	**	**	**	ns
GS	ns	ns	***	ns	ns	***	ns	***
MS × GS	ns	ns	ns	ns	ns	ns	**	ns

Legend: ns, **, and *** non-significant or significant at *p* ≤ 0.05, *p* ≤ 0.001, and 0.001, respectively. Different letters within each column indicate significant mean differences according to Student’s *t*-test (*p* = 0.05). All data are expressed as mean ± standard error, n = 4.

**Table 5:** Effect of species and growing system on organic acid content.

Treatments	Malate	Oxalate	Citrate
	g kg^−1^ DW		
Microgreen species (MS)			
Mibuna	22.74 ± 5.01	2.22 ± 0.09	10.13 ± 1.2
Pak Choi	14.06 ± 4.74	2.03 ± 0.08	7.65 ± 1.17
Growing system (GS)			
With Substrate	29.08 ± 2.79 a	2.11 ± 0.08	11.89 ± 0.56 a
Without Substrate	7.71 ± 0.87 b	2.14 ± 0.08	5.88 ± 0.49 b
MS	ns	ns	ns
GS	***	ns	***
MS × GS	**	ns	ns

Legend: ns, **, and *** non-significant or significant at *p* ≤ 0.01 and 0.001, respectively. Different letters within each column indicate significant mean differences according to Student’s *t*-test (*p* = 0.05). All data are expressed as mean ± standard error, n = 4.

## Data Availability

All data supporting the findings of this study are included in the article and its Supplementary Material (Excel file). Further inquiries can be directed to the corresponding authors.

## Supplementary Materials

The following supporting information can be downloaded at https://www.mdpi.com/article/10.3390/plants15030465/s1. All experimental data subjected to analysis of variance (ANOVA) are provided in the Supplementary Excel file Raw_Data_Ciriello_et_al_Plants_MDPI.xlsx.
